# A Scalable Network Model for Electrically Tunable Ferroelectric Domain Structure in Twistronic Bilayers of Two-Dimensional Semiconductors

**DOI:** 10.1021/acs.nanolett.1c04210

**Published:** 2022-02-07

**Authors:** Vladimir V. Enaldiev, Fabio Ferreira, Vladimir I. Fal’ko

**Affiliations:** † University of Manchester, School of Physics and Astronomy, Oxford Road, Manchester M13 9PL, United Kingdom; ‡ National Graphene Institute, University of Manchester, Booth Street East, Manchester M13 9PL, United Kingdom; ¶ Kotelnikov Institute of Radio-engineering and Electronics of the RAS, Mokhovaya 11-7, Moscow 125009, Russia; § Henry Royce Institute, University of Manchester, Booth Street East, Manchester M13 9PL, United Kingdom

**Keywords:** Ferroelectrics, twistronic heterostructures, domain wall network, van der Waals heterostructures

## Abstract

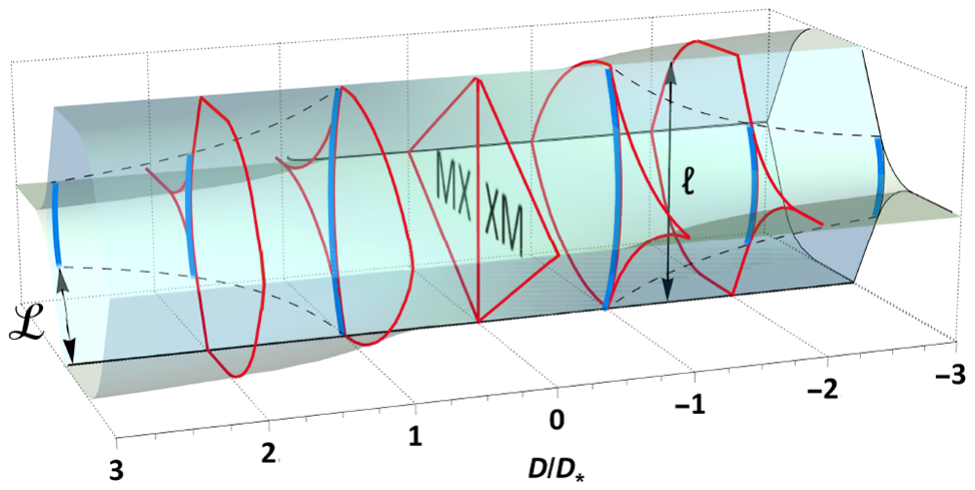

Moiré structures in small-angle-twisted bilayers of two-dimensional (2D) semiconductors with a broken-symmetry interface form arrays of ferroelectric (FE) domains with periodically alternating out-of-plane polarization. Here, we propose a network theory for the tunability of such FE domain structure by applying an electric field perpendicular to the 2D crystal. Using multiscale analysis, we derive a fully parametrized string-theory-like description of the domain wall network (DWN) and show that it undergoes a qualitative change, after the arcs of partial dislocation (PD) like domain walls merge (near the network nodes) into streaks of perfect screw dislocations (PSD), which happens at a threshold displacement field dependent on the DWN period.

## Introduction

Two-dimensional material twistronics, fueled by discoveries of new phenomena in twisted graphene bilayers^1−10^ and trilayers,^11−16^ has recently expanded onto a broader range of van der Waals systems.^17−23^ In general, twistronic structures are associated with geometrical moiré patterns: a periodic variation of local stacking of the two layers. In long-period moiré patterns, characteristic for small-angle-twisted bilayers, the areas of energetically preferential stacking expand into mesoscale domains,^24−27^ embedded into a domain wall network (DWN). In particular, by assembling a homobilayer of two inversion-asymmetric honeycomb monolayers (hBN or transition metal dichalcogenides (TMD)) with parallel orientation of their unit cells, one obtains an array of triangular domains for which chalcogen atoms (X) in one layer are vertically aligned with metal atoms (M) of the neighboring layer.^27−30^ Below, we label the two type of domains with chalchogen-over-metal and metal-over-chalcogen stackings XM and MX, respectively. These domains possess lattice structure of 3R-TMD polytype with broken mirror and inversion symmetries^31−36^ and also manifest an out-of-plane ferroelectric (FE) polarization,^37−39^ with opposite direction in the mirror-symmetry-related XM and MX domains.^28,30^

**Figure 1:**
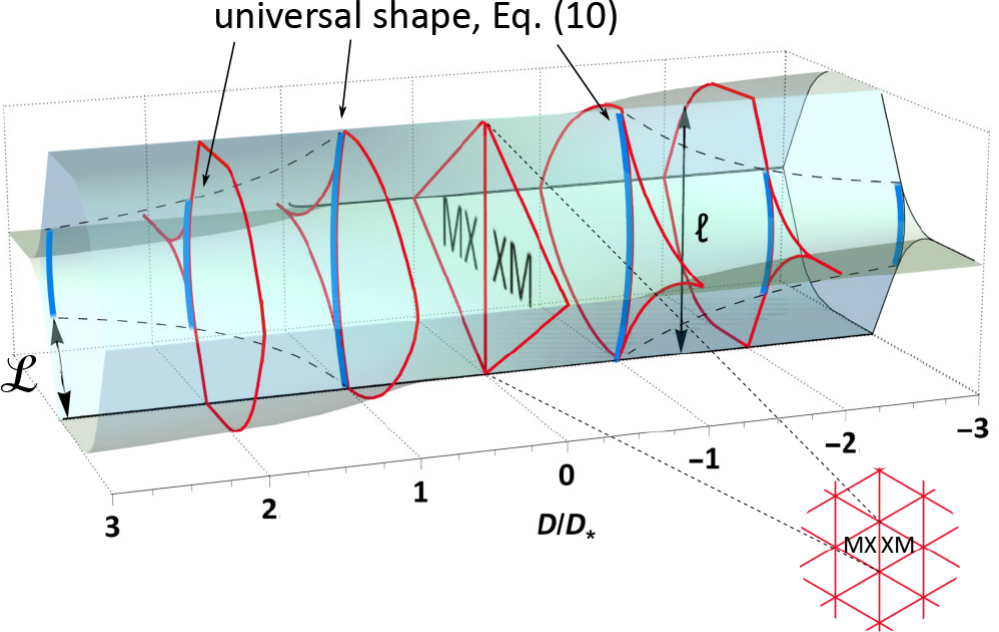
Triangular XM/MX DWN (inset) in twisted TMD bilayers with FE domains areas varied by an out-of-plane displacement field, *D*. At *D* ≥ D_*_, XM/MX domain walls merge into streaks of perfect screw dislocations separating—near the network nodes—domains with the same FE polarization. The remaining arcs of XM/MX domain walls have a universal shape, scaled with D_*_/*D* ratio.  is a period of DWN. Displayed evolution of the moiré supercell of the twisted TMD bilayers in the displacement field constitutes the key result of our Letter.

The coupling between the out-of-plane FE polarization and an externally controlled displacement field, *D*, varies the energies of XM and MX, changing the ratio between their areas and leading to the deformation of DWN. Here, we offer a generic theory for the field-tunable FE domain structure in twistronic TMD bilayers, fully quantified with the help of multiscale modeling approach^28,39−41^ for MX_2_-bilayers (M = Mo,W; X = S,Se). Lattice reconstruction in TMD bilayers without electric field has been studied before in refs (28−30) within different approaches consistent in formation of the triangular domains close to zero twist angles. The predictions have been experimentally verified in refs (26 and 27). The focus of this study is on the influence of external electric field on the domain structure for which we develop a string-theory like model for the DWN. To the best of our knowledge the issue have not been addressed before. To anticipate, the main finding of this study is that there are two regimes of DWN evolution driven by the out-of-plane electric field, separated by the threshold displacement field value, . For weak fields |*D*| < D_*_, the continuously deforming domain walls retain their partial dislocation character, however, above the threshold, |*D*|/D_*_ ≥ 1, pairs of partial dislocations (PD) form streaks of perfect (full) screw dislocations (PSD, of length ) near the network nodes. This evolution is illustrated in Figure 1, where the arcs of XM/MX domain walls with a universal shape merge and split apart upon the variation of *D*.

The scenario of the DWN transformation in Figure 1 is a result of the following analysis. First, we use density functional theory (DFT) to quantify the coupling of FE polarization of an asymmetric TMD interface to an external out-of-plane displacement field, *D*. By taking into account the resulting coupling in the competition between the interlayer adhesion and elastic strain in the layers, which has been used^28^ and tested^27^ earlier in the studies of mesoscale lattice relaxation in TMD bilayers, we derive an effective theory, formulated in terms of the DWN deformations. Finally, we find an analytical solution for such a “string-like” theory, which has a universal form scaling with the *D*/D_*_ ratio.

## Ab Initio Modeling of FE Bilayers in the Out-of-Plane Electric Field

**Figure 2:**
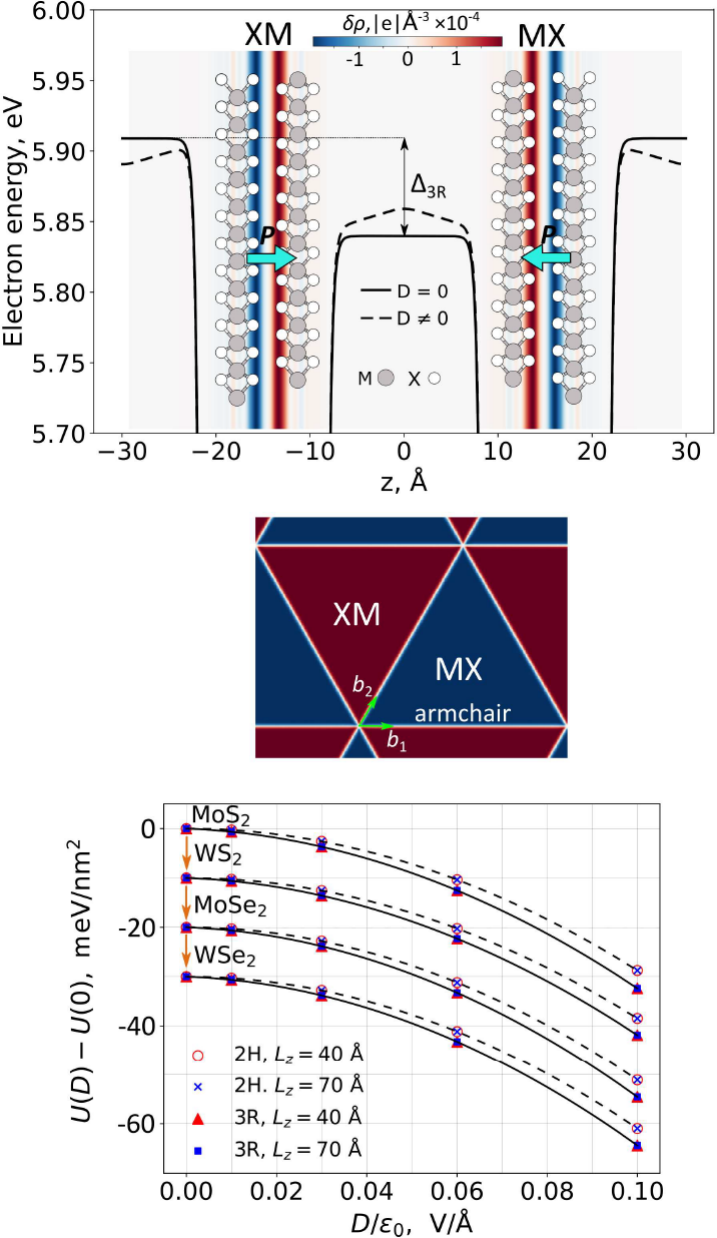
Top: Solid (dashed) line shows potential energy of electrons computed in DFT for a double supercell structure of TMD bilayers with 3R stacking (here, MoS_2_) with and without external displacement field. A map, superimposed on the lattice structure, visualizes FE charge density, *δρ*(averaged over the unit cell are). The two twin bilayers in the expanded supercell, used in the DFT modeling, represent the actual lattice structure of MX and XM domains in the reconstructed moire superlattice of a TMD bilayer, which at *D* = 0 have a triangular shape (middle panel). Bottom: Red and blue symbols show DFT-computed energies of 3R/2H-bilayer per unit cell as a function of a displacement field at vacuum spacer lengths *L*_*z*_ = 40 and 70 Å, respectively. Solid/dashed lines demonstrate their fit with eq 1 (for 2H *P* = 0) with the results gathered in Table 1. For clarity, data for WS_2_, MoSe_2_, and WSe_2_ bilayers are vertically shifted consecutively by half of the interval between the closest ticks.

Here, we use two methods to carry out DFT calculations for 3R-bilayers, with the coinciding results. In the first method, we use Quantum ESPRESSO (QE)^42,43^ to construct a supercell with a pair of mirror-reflected (MX and XM) bilayers, separated by a large vacuum gap, as shown in Figure 2. This choice of the structure eliminates an issue with periodic boundary condition for potential of the FE charges.^39^ In the second method we construct a single 3R-bilayer and use Coulomb truncation in the out-of-plane direction^44^ with QE and dipole-correction with VASP.^45^ An example of the computed charge transfer, *δρ*, between the layers of an individual bilayer (which integral, ∫ *zδρ*(*z*) d*z* ≡ *P*, determines the areal density of the FE dipole moment) and a potential drop, Δ, across the double charge layer, is shown in Figure 2. To mention, the computed values of *P* and Δ are related as *P* = ϵ_0_Δ, in agreement with the earlier studies of 3D bulk FE materials.^46^

The displacement field, accounted for by adding a triangular potential, −*D*|*z*|/ϵ_0_, to the input pseudopotentials, produces a shift, *δU* ≡ *U*(*D*) – *U*(0) of the bilayer energy. The latter includes a linear term, accounting for the FE polarization, and a quadratic downward shift because of the dielectric out-of-plane polarizability of the material:
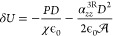
1Here, we parametrize the *P*–*D* coupling by a dimensionless parameter χ and the out-of-plane dielectric polarizability by α_*zz*_^3R^ (where  is the unit cell area of a TMD monolayer). The DFT-computed energies of bilayers of four different TMDs are shown in Figure 2: in this computation, we used two different supercell periods (*L*_*z*_ = 40 Å and *L*_*z*_ = 70 Å, which set the length of the vacuum spacer). Using eq 1, we find the polarizability values, which are very close to polarizability, α_*zz*_^2*H*^, computed for the inversion-symmetric 2H bilayer^a^. When recalculated per monolayer, these values also agree with the separately computed monolayer polarizability, α_*zz*_ (α_*zz*_ = (1/2)α_*zz*_^3*R*^ = (1/2)α_*zz*_^2*H*^, see in Table 1). This confirms that the dielectric response of a wide band gap van der Waals materials is determined by the intralayer polarization of the constituent atoms. These values enabled us to estimate the *z*-axis dielectric susceptibility of bulk TMD crystals,^47−49^, arriving at the values in the range of ϵ_*zz*_ ∼ 6–7.5, listed in Table 1.

**Table 1:** Dielectric and FE Parameters for Various TMDs^a^

	α_*zz*_^3R^ (Å^3^)	α_*zz*_^2*H*^ (Å^3^)	α_*zz*_ (Å^3^)	ϵ_*zz*_	χ	Δ (mV)	Δ_*a*_ (mV)	*q* (nm^–1^)
MoS_2_	89.89	89.80	44.46	6.45	1.03	68	16.4	22.152
	89.93	89.85	44.48	6.48	1.02			
WS_2_	88.43	88.37	43.95	5.95	1.00	62	15	22.598
	88.43	88.37	43.94	5.95	1.00			
MoSe_2_	105.32	105.22	52.29	7.65	1.05	66	15.7	20.520
	105.31	105.25	52.24	7.67	1.07			
WSe_2_	104.37	104.33	52.03	7.39	1.04	65	15.3	20.953
	104.32	104.28	51.97	7.32	1.07			

a Left: Polarizability (α_*zz*_) (used to estimate the dielectric permittivity ϵ_*zz*_ of 3D-bulk TMDs with experimentally determined^50,51^ interlayer distances, *d*), and FE coupling parameter (χ) for various 3R-TMD bilayers. Right: FE potential drop parameters in eq 3. For α_*zz*_^3R^, χ and ϵ_*zz*_ we show the values obtained with QE (top row) and, for comparison, with VASP (bottom row). For QE, we used full-relativistic ultrasoft pseudopotentials and a plane-wave cutoff energy of 70 Ry. For VASP, we used PAW full-relativistic pseudopotentials and 60 Ry cutoff (with a 13 × 13 × 1 *k*-point grid and a Perdew–Burke–Ernzerhof (PBE)^52^ exchange-correlation functional for both QE and VASP).

Despite a substantial polarizability of monolayers, the data in Figure 2 are described well by eq 1 with χ≈ 1, pointing toward a decoupling of the interlayer FE charge transfer from the intralayer dielectric polarizability. Moreover, by comparing the DFT-computed values of the double-layer potential drop Δ to the FE coupling with the displacement field, *D*, in eq 2, we find that the linear in *D* energy shift in MX and XM domains (which have opposite out-of-plane FE polarization, ±*P*, and voltage drop across the double charge layer, ±Δ) can be described very well as
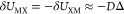
2Expression 2 naturally comes when assuming a local dielectric permittivity in a continuum medium approximation. Indeed, suppose the FE charges, with plane-averaged density *δρ*(*z*), are placed in the medium with local dielectric permittivity ϵ_*zz*_(*z*). From the Poisson equation, ∂_*z*_(ϵ_*zz*_(*z*)∂_*z*_φ(*z*)) = −*δρ*(*z*)/ϵ_0_, and electroneutrality condition, ∫_–∞_^+∞^*δρ*(*z*) d*z* = 0, we express the potential drop across the layer of charges as Δ = ∫_–∞_^+∞^ ∂_*z*_φ(*z*) d*z* = ∫∫_*z*<*z*′_ d*z* d*z*′*δρ*(*z*′)/ϵ_*zz*_(*z*)ϵ_0_. At the same time, interaction energy of these charges with uniform external out-of-plane displacement field (related to local electric field as *D* = ϵ_0_ϵ_*zz*_(*z*)*E*(*z*)) reads as *δU* = −∫_–∞_^+∞^ d*zδρ*(*z*) ∫_–∞_^*z*^ d*z*′*D*/ϵ_*zz*_(*z*′)ϵ_0_ = −*D*∫∫_*z*<*z*′_ d*z* d*z*′*δρ*(*z*′)/ϵ_*zz*_(*z*)ϵ_0_ ≡ −*DΔ*, where we changed variables, *z* ↔ *z*′, after the first step. Below, we will use this coupling to model tunability of the DWN by displacement field.

## Mesoscale Model for Lattice Reconstruction

**Figure 3:**
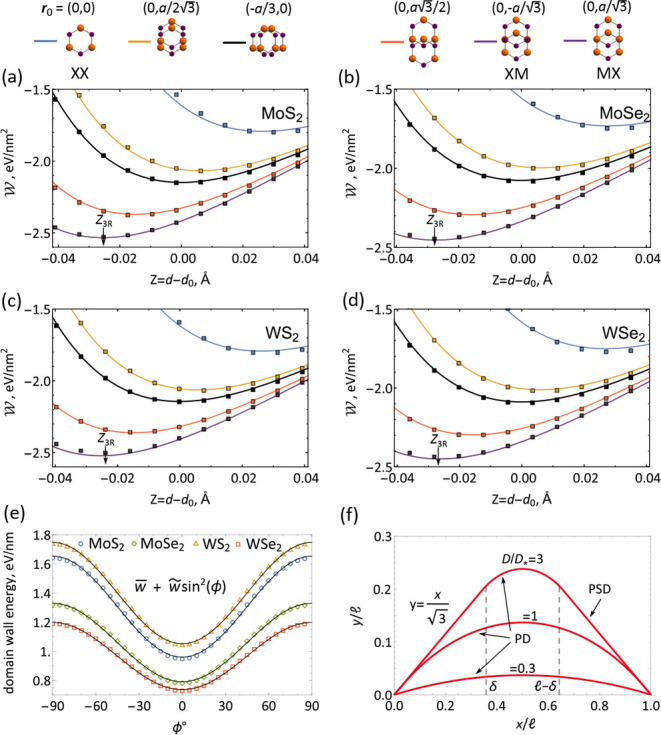
(a–d) TMD bilayer adhesion energies for various offsets and interlayer distances, *Z* = *d* – *d*_0_, counted from the optimal distance, *d*_0_, of stacking configurations averaged adhesion energy. *Z*_3R_ labels interlayer distance used to evaluate the FE parameters in 3R (MX and XM) TMD bilayers. Details of DFT data (square dots) are described in ref (28). Insets show stackings with corresponding in-plane offsets between the layers. (e) Orientation dependence of partial dislocation energy per unit length calculated in ref (28) and fitted with *w* + *w̃* sin^2^ ϕ, where values of parameters are listed in Table 2. ϕ = 0 corresponds to the armchair direction. (f) Evolution of domain wall shape with displacement field, given by solution of eq 5 for *D* < D_*_ and *D* ≥ D_*_.

Mesoscale model for lattice reconstruction is formulated in terms of the bilayer energy dependence on local stacking of the two layers and their strain,^27,28,40^ incorporated via an interlayer offset, ***r***_0_(***r***) = *θẑ* × ***r*** + ***u***^(t)^ – ***u***^(b)^, which varies across the superlattice, as prescribed by a small-angle, θ, misalignment between the top (t) and bottom (b) crystals and their elastic deformations, ***u***^(t/b)^. Locally, stacking determines the interlayer distance, which corresponds to the minimum of the stacking-dependent adhesion energy, , quantified for the four TMDs using DFT modeling and displayed in Figure 3. For convenience, the interlayer distance (*d*) dependence of the computed  is shown as a function of *Z* = *d* – *d*_0_, counted from the minimum (at *d* = *d*_0_) of the offset-averaged adhesion energy, which coincides with  (one of stacking configurations shown in Figure 3). As a result, energy density, characterizing the relaxation functional, reads
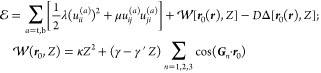
3Here, the first term accounts for strain, *u*_*ij*_^(*t*/*b*)^ = (1/2)(∂_*i*_*u*_*j*_^(*t*/*b*)^ + ∂_*j*_*u*_*i*_^(*t*/*b*)^) (λ and μ are the monolayer elastic moduli). The second term describes adhesion energy between top and bottom layers, where κ determines curvature of the adhesion energy of 3R-stacked bilayers, which characterizes frequency of layer breathing mode,^28^ and ***G***_1,2,3_ is the first star of reciprocal lattice vectors of TMD monolayer, |***G***_1,2,3_| = 4π/*a*√3 (for details, see refs.^27,28,40^). The last term is responsible for the energy shift due to the external field.

The next steps in the analysis require choosing an optimal interlayer distance between the two monolayers, which depends on the encapsulation environment of the bilayer. For a bilayer in vacuum or encapsulated into a soft flexible matrix with a weaker adhesion to TMD than the interlayer adhesion , we find from eq 3 that, locally, . For a strong substrate–TMD coupling, the monolayers would remain flat, and the optimal interlayer distance would be *Z* = 0 across the entire moiŕe pattern. For either of these two cases, we substitute the corresponding choice of *Z* in eq 3, and also into the local voltage drop across the double layer of charge (in the last term in eq 3) corresponding to the local stacking configuration, which form has been established earlier:^39^
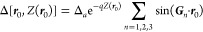
For XM and MX stackings, this gives Δ ≡ Δ(***r***_0_^MX^) = −Δ(***r***_0_^XM^), which values, taken from the recent ab initio simulations,^28,41^ are listed in Table 1.

## Network Model

While the minimization of the energy functional in (3) enables one to find all mesoscale details of the lattice adjustments of the two crystalline planes of the bilayer, it is more practical to use another method for the analysis of small-angle (θ ≲ 0.5°) twisted bilayers. This is because, in the latter case, most of the areas of the moiré pattern is occupied by the homogeneous MX and XM stacking domains with a characteristic size of , whereas the deformations are concentrated inside narrow domain walls, which are only few nanometers in width.^27,28^ Then, we define the DWN energy
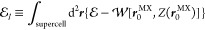
by subtracting the energy of a uniform MX-stacked bilayer at *D* = 0 from  in eq 3. This reduces the problem to the analysis of , where we can treat each domain wall as a string, characterized by energy per unit length dependent on a crystallographic orientation of the domain wall axis,^28^ with, as shown in Figure 3e, a pronounced minimum at the armchair direction in the TMD crystal.

A finite displacement field acts as an external drive for increasing areas of domains with the preferential FE polarization. This leads to the bending of the PD domain walls, hence, increasing their energy due to their elongation and the PD axis deviation from the armchair axis. The exact form of the deformed DWN can be found by solving a “string theory” model, expressed in terms of a deflection, *y*(*x*), of PD segments from the closest armchair direction, formalized using an energy functional,
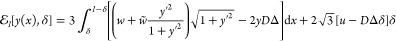
4Here, the first term in the integral describes the orientation-dependence of the PD energy, *w* + *w̃* sin^2^ ϕ, and its stretching, accounted by a factor  with ϕ standing for an angle between the dislocation axis and the closest armchair direction in the crystal, so that sin^2^ϕ = *y*′^2^/(1 + *y*′^2^). Two stiffness parameters, *w* and *w̃*, were determined using the data from ref (28), see Table 2 for their values for various TMDs. The second term in the integral stands for the energy gain from a bigger area of the energetically preferential FE polarization domain. The last two terms in  accounts for two PDs merging, near each DWN node, into streaks of PSDs with a length  (projected onto 0 ≤ *x* ≤ δ and  intervals), which energetically preferable orientation is along zigzag axis in the crystal and energy per unit length is *u* (Table 2).

**Table 2:** Elastic Moduli, Adhesion, and Partial Dislocation Energy Density Parameters for the Studied TMD Bilayers

	γ (eV/nm^2^)	γ′ (eV/nm^3^)	κ (eV/nm^4^)	μ (N/m)	λ (N/m)	*a* (nm)	*d*_0_ (nm)	*w* (eV/nm)	*w̃* (eV/nm)	*u* (eV/nm)
MoS_2_	0.189	5.634	214	70.9	83.2	0.316	0.636	0.96	0.69	2.24
WS_2_	0.212	6.327	213	72.5	52.5	0.315	0.638	1.05	0.69	2.45
MoSe_2_	0.1975	5.707	189	49.6	42.3	0.329	0.670	0.79	0.54	1.84
WSe_2_	0.1514	4.381	190	48.4	29.7	0.328	0.671	0.74	0.46	1.69

Using variational principle, we obtain an equation for the shape of each string,
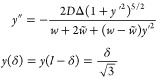
5where δ appears as an additional variable. Note that zero values of *y*(*x*) at the network nodes requires vanishing of derivative in the middle of interval, that is, providing a symmetrical shape of the PD, with .

For a small *D*, energetically favorable domains grow due to bending of PDs, with their ends fixed at network nodes, δ = 0. This elastic stretching has no threshold in *D* and the string form is described by^b^
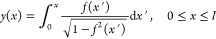
6where *f*(*x*) is a real root of
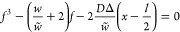
7Note that  and that *f*(*x*) ≡ 0 for *D* = 0.

**Figure 4:**
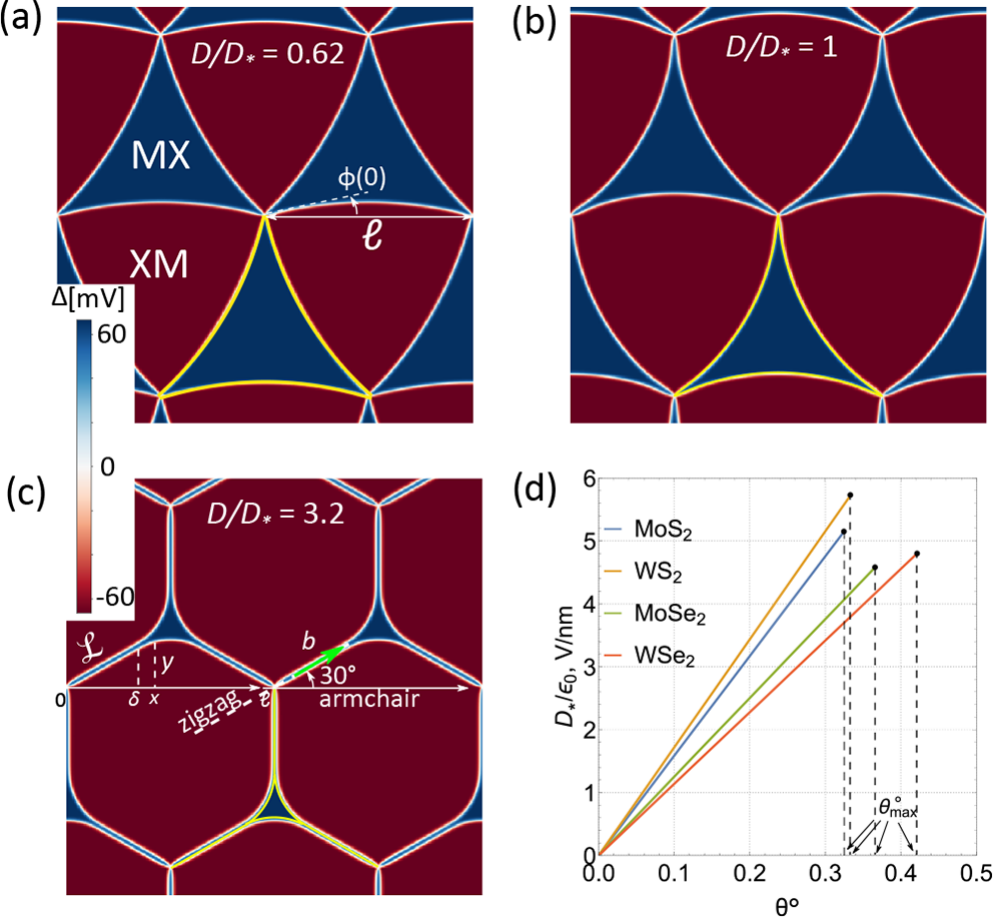
(a–c) Maps of the interlayer potential drop, Δ in MoS_2_ bilayer with a θ = 0.1° twist, computed by the minimization of energy in eq 3 for various values of *D* below and above the threshold. These maps display the deformation of DWN from a triangular web of partial dislocations—see Figures 1 and 2, with a  period. Yellow lines are the streaks of PSD and arcs of PD described by eqs 6–10. (d) Dependences of threshold electric fields on twist angles terminating at values corresponding to electric breakdown of the TMD bilayers.

Bending, described by eq 6, takes a new qualitative form after each pair of PDs near the network nodes  touch each other, see Figure 4b. This condition corresponds to ϕ°(0) = −ϕ°(*l*) = 30° (i.e., ), which, together with eq 6 determines a threshold displacement field
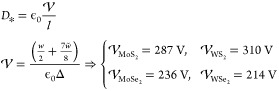
8

It is important to note that pairs of touching PDs cannot annihilate because a sum of their Burgers vectors ***b***_1_ + ***b***_2_ = ***b***(***b***_1_=*a*(1/√3,0), ***b***_2_ = *a*(1/2√3,1/2), ***b*** = *a*(√3/2,1/2)) corresponds to the Burgers vector (|***b***| = *a*) of a PSD, required to maintain an overall twist between two monolayers in the bilayer. This means that they actually form streaks of PSDs, which lengths grow with a further increase of the electric drive, as
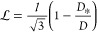
9The latter scaling law follows from the solution of eq 5 on the interval  with the boundary conditions, , where  is a projection of  onto the *x*-axis. Hence, above the threshold, DWN is composed of (A) PSD streaks near each network node aligned with zigzag directions in TMD, and separating the adjacent expanded energetically favorable 3R-domains and (B) PD arcs splitting up at the PSD ends, *x* = δ and , and touching each other at the splitting points.

To find the shape of the remaining PD arcs for any *D* > D_*_, we rewrite eq 5 in dimensionless coordinates, *x̃* and *ỹ* defined by , ,
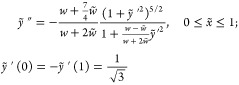
Its solution has the same structure for any *D* ≥ D_*_
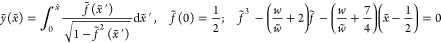
10describing the universal shape of partial dislocation arcs, scaled by the ratio D_*_/*D*, as shown in Figure 3f.

To validate the accuracy of the obtained analytical solution of the network model, we also performed the mesoscale lattice relaxation for a MoS_2_ bilayer with a 0.1° interlayer twist (corresponding to ), by minimizing the full energy functional in eq 3. For this, we analyzed the Euler–Lagrange equations for the deformation fields ***u***^(t/b)^, obtained by applying variational principle to . We solved those numerically, seeking for periodic (with the period ) solutions on a sufficiently dense grid using interior point method implemented in GEKKO Optimization Suite package.^53^ The computed fields were used to obtain the local values of the interlayer offset, ***r***_0_(***r***) = *θẑ* × ***r*** + ***u***^(*t*)^–***u***^(*b*)^, which we substituted into eqs 3 to find the interlayer distance, *Z*, and to map the interlayer potential drop across the domain structure. The results for the latter quantity are shown in Figure 4a–c for both pre- and post-threshold displacement fields, displaying a close agreement with the analytical solution of the network model shown by yellow lines.

Finally, in Figure 4d, we plot dependences of the threshold displacement fields on the twist angles, , demonstrating feasibility of the scaling regime (*D* > D_*_) for the considered twisted TMD bilayers at the small twist angles (. The upper limits for the maximal twist angles (or the shortest periods) in the dependences are set by electric breakdown fields of the TMD bilayers estimated using band gap^54^*E*_g_ as 2*E*_g_/*ed*(1 + ϵ_*zz*_^–1^), where *e* is elementary charge. We note that for TMD bilayers encapsulated in hexagonal boron nitride (hBN) the magnitudes of electric breakdown fields for TMD is smaller than that for hBN, as the latter possesses larger band gap and smaller interlayer distance. Therefore, electric breakdown of TMD bilayers imposes stricter bounds for range of applied electric fields.

## Conclusion

To conclude, the developed effective network model gives an efficient description of the long-period domain structure in small-angle-twisted TMD bilayers with ferroelectric interface and its deformation by an out-of-plane electric field. Its analytical solution in eqs 6–10 gives a simple tool for the interpretation of experimentally measured variations of the domains’ shapes, such as in the recently studied bilayers with ferroelectric properties.^32−36^

## Footnotes

a For monolayers and 2H-bilayers, we used the Coulomb truncation in the out-of-plane direction.^44^

b At small *D* (when *y*′^2^ ≪ 1), the approximate solution of eq 5 is given by parabola: .
